# Assessing the Views and Needs of People at High Risk of Gestational Diabetes Mellitus for the Development of Mobile Health Apps: Descriptive Qualitative Study

**DOI:** 10.2196/36392

**Published:** 2022-07-08

**Authors:** Beibei Duan, Zhe Liu, Weiwei Liu, Baohua Gou

**Affiliations:** 1 School of Nursing Capital Medical University Beijing China; 2 Beijing Youyi Hospital Capital Medical University Beijing China

**Keywords:** gestational diabetes mellitus, high-risk groups, mobile health, mHealth, applications, user-centered design, qualitative research

## Abstract

**Background:**

Early prevention of gestational diabetes mellitus (GDM) can reduce the incidence of not only GDM, but also adverse perinatal pregnancy outcomes. Moreover, it is of great significance to prevent or reduce the occurrence of type 2 diabetes. Mobile health (mHealth) apps can help pregnant women effectively prevent GDM by providing risk prediction, lifestyle support, peer support, professional support, and other functions. Before designing mHealth apps, developers must understand the views and needs of pregnant women, and closely combine users’ needs to develop app functions, in order to better improve user experience and increase the usage rate of these apps in the future.

**Objective:**

The objective of this study was to understand the views of the high-risk population of gestational diabetes mellitus on the development of mobile health apps and the demand for app functions, so as to provide a basis for the development of gestational diabetes mellitus prevention apps.

**Methods:**

Fifteen pregnant women with at least one risk factor for gestational diabetes were recruited from July to September 2021, and were interviewed via a semistructured interview using the purpose sampling method. The transcribed data were analyzed by the traditional content analysis method, and themes were extracted.

**Results:**

Respondents wanted to develop user-friendly and fully functional mobile apps for the prevention of gestational diabetes mellitus. Pregnant women's requirements for app function development include: personalized customization, accurate information support, interactive design, practical tool support, visual presentation, convenient professional support, peer support, reasonable reminder function, appropriate maternal and infant auxiliary function, and differentiated incentive function.These function settings can encourage pregnant women to improve or maintain healthy living habits during their use of the app

**Conclusions:**

This study discusses the functional requirements of target users for gestational diabetes mellitus prevention apps, which can provide reference for the development of future applications.

## Introduction

Gestational diabetes mellitus (GDM) refers to the first occurrence of abnormal glucose tolerance of varying degrees during pregnancy, which is one of the most common complications of pregnancy [1]. Due to differences in diagnostic criteria and race, the global incidence of GDM is between 1% and 25% [2]. A previous study has shown that the incidence of GDM in China is 14.8%, and with the adjustment of the fertility policy and the increase in elderly people and obese pregnant women, the incidence of GDM shows a trend of increasing every year [3,4]. GDM brings serious threats and challenges to both maternal and infant health. GDM can increase the risks of pregnancy hypertension, abortion, polyhydramnios, premature delivery, dystocia, and cesarean section [4-6]. Women who have a history of GDM have a 50%-73% risk of recurrence of GDM when they get pregnant again and a 10-fold higher risk of developing type 2 diabetes in 5 to 10 years after delivery, and the risk of cardiovascular disease is also higher in these women than in normal women [7]. Compared with the offspring of healthy women, the offspring of GDM patients have higher risks of fetal malformation, macrosomia, large for gestational age, hyperinsulinemia, neonatal hypoglycemia, pathological jaundice, and respiratory distress syndrome [8,9]. In addition, these children have increased risks of obesity, abnormal glucose tolerance, and type 2 diabetes when they become adults, bringing heavy economic burden to countries around the world [10]. Therefore, early preventive measures for pregnant women at high risk of GDM are of great significance in reducing the incidence of GDM, reducing adverse perinatal pregnancy outcomes, and preventing or reducing the occurrence of type 2 diabetes.

Mobile health (mHealth) refers to medical treatment and health management through mobile devices, such as mobile phones, patient health data monitoring devices, palm computers, and other wireless devices [11]. Mobile information technology can bridge the communication between medical service providers and users, helping doctors understand the health status of users and providing clinical decision support, so as to achieve remote diagnosis and treatment. At the same time, users’ needs for self-management support can be met to improve their compliance and self-management behaviors [12-14]. In addition, mobile information technology can greatly save users’ time and transportation costs, relieve the burden of medical treatment faced by hospitals and community health service institutions, and make the limited medical service resources the most effective. Therefore, mHealth has significant potential in the medical and health fields [15,16].

As one of the main forms of mHealth management, mHealth apps refer to health service platforms that use smartphones, tablets, and other mobile devices as terminals and rely on mobile internet technology to provide services for patients and medical staff [17]. With the popularity of mobile devices, more than 500 million smartphone users worldwide use mHealth apps for health management [18]. Due to their unique biofeedback function, mHealth apps can carry out real-time health assessment and provide feedback on patients’ health status, which can be considered beneficial for medical staff to implement personalized and precise health management for patients, and can have good application prospects [19]. At present, mHealth apps have been widely used in the prevention and health management of diabetes patients and have achieved satisfactory results [20]. However, currently, apps are mostly developed for commercial needs and purposes, the needs of patients are seldom taken into account during development, and few patients are invited to participate in the design process of apps [21-23]. Studies have shown that lack of interest in apps, fees, and fear of personal information disclosure are the main reasons that hinder users from downloading and sticking with apps [24]. Therefore, when developing mHealth apps, it is necessary to take users as the center, pay attention to users’ preferences and needs for using apps, and design apps that can meet users’ needs, so as to improve users’ experiences, increase users’ stickiness in using apps, and improve patients’ compliance with self-management.

User-centered design (UCD), which is part of human-computer interaction, takes user needs into account at every stage of product development. It is an important step and a relatively mature design method for building application programs [25]. Its core is to understand users and build their psychological model into system functions, so as to provide customized high-quality nursing services for each user more effectively [26]. In the process of app development, UCD gives priority to the needs of users, which can improve the stickiness and autonomy of users, and bring positive emotional experience to users, so as to meet the needs, preferences, and goals of users, and improve the quality of apps. The World Health Organization recommends that it be integrated into the whole process of an mHealth intervention to ensure the effectiveness of the intervention [27-30]. In recent years, UCD technology has been applied in the development of mHealth apps and has achieved certain effects in the areas of lifestyle intervention for patients with chronic diseases [25]. Studies have shown that most health management apps are not designed with user-centered methods, leading to poor usability and less use of these apps [31].

At present, some apps for GDM health management have been developed by scholars, but few studies have applied mHealth apps to GDM prevention management [32]. As mentioned above, understanding the usage preferences of mHealth apps among people at risk of gestational diabetes is crucial for the development of GDM prevention apps. However, there is no evidence that people at risk of gestational diabetes have a preference for mHealth apps. Qualitative research methods can dig deep into the inner needs of pregnant women. Therefore, the purpose of this study was to use a user-centered qualitative research method to interview pregnant women at high risk of GDM in early pregnancy, in order to explore the needs and preferences of these pregnant women for the functions and design of mHealth apps. The results can provide a reference for the design and development of mHealth apps for the prevention of gestational diabetes, and can help to adjust intervention measures, improve the acceptance and effectiveness of app use by pregnant women, and improve user engagement.

## Methods

### Study Design

In this study, the descriptive qualitative research method was adopted and the semistructured in-depth interview method was used to collect data. According to the purpose of the study, the interview outline was designed based on previous experience and reference to relevant literature. Before the formal interview, 2 pregnant women were preinterviewed, and the interview outline was modified appropriately according to the results of the preinterview analysis. The formal interview outline has been presented in Textbox 1.

**Semistructured interview guide used in this study.**

**Guide**

1. Which mobile health management apps have you used before? What features do you find particularly useful in these apps? Or which features are not very useful and could be improved further?

2. If an app for gestational diabetes prevention is developed to assist your daily health management, what functions do you expect it to have?

3. What are your requirements for the interface design of an app?

4. What other requirements that you would like us to consider for developing an app for gestational diabetes prevention?

### Participants and Recruitment

The maximum difference sampling strategy was adopted to select pregnant women with different age, occupation, parity, and gestational diabetes risk factors as far as possible for the purpose of sampling, so as to ensure diversity among the respondents. From July to September 2021, in the obstetrics clinic of a third-class hospital in Beijing, a researcher publicized the project to pregnant women, who made an appointment to establish routine health records, and invited pregnant women to share their experiences and opinions in depth in the form of face-to-face interviews. The inclusion criteria were as follows: (1) at least one risk factor for gestational diabetes (eg, advanced maternal age, overweight or obesity, family history of diabetes, history of GDM, history of macrosomia, and impaired fasting glucose); (2) gestational age <12 weeks; (3) experience of using sports apps, maternal and child health apps, or other health management apps; (4) good communication skills; and (5) informed consent and voluntary participation in this study. The exclusion criteria were as follows: (1) age <18 years and (2) presence of mental disorders. The interviews in this study were stopped when no new topics appeared, that is, the data were saturated. A total of 16 pregnant women were interviewed, and 1 of the women dropped out because she had something else to do halfway. Finally, 15 women participated in semistructured interviews (participant #1 to participant #15). The mean age of the respondents was 32 (SD 3.44) years, and the mean gestational age was 6.8 (SD 0.74) weeks. There were 12 first-time pregnancies and 3 second-time pregnancies. One of the pregnant women was a stay-at-home mother, and the other 14 were from different occupations. Six had 2 or more risk factors, and the remaining 9 had only 1 risk factor. Among the 15 pregnant women, 1 was of Hui nationality and the other 14 were of Han nationality. With regard to the education level, 3 had junior college or below education, 10 had undergraduate education, and 2 had graduate or above education. In terms of cost, 1 of the 15 pregnant women was self-paid, while the rest were covered by medical insurance.

### Data Collection

Face-to-face interviews were conducted. Each interview was conducted in a quiet outpatient lounge with no third person to disturb. The interview duration was 20-40 minutes. Before the interview, interviewers introduced to interviewees the definition, harm, and intervention status of gestational diabetes. The purpose and significance of the interviews were also informed. Interviewees were told that their privacy would be protected by the researchers. The interview was recorded with the consent of the interviewees. The interview was conducted according to the interview outline. During the interview, the interviewers carefully listened to the statements; appropriately responded to them with questioning, repetition, clarification, response, and summary; encouraged participants to fully express their ideas; avoided inductive questioning; and timely recorded the key information of the interview. At the same time, they paid attention to observe and record the interviewees’ nonverbal information, such as a pause, a smile, body language, and mood change. After each interview, a reflective diary was written to reflect on the problems in the interview and correct them in the next interview. After the interview, the interviewees were thanked for their participation and were informed of the possibility of contacting them again for further information.

### Data Analysis

Data collection and analysis were conducted simultaneously. After each interview, interviewers listened to the original materials repeatedly. The materials were transcribed within 24 hours. Uncertainties were clarified in time in combination with on-site notes. The traditional content analysis method was used for data analysis. The final transcribed text was merged into a single text by topic. Interviewers read the text several times to get a sense of the whole text. Selection criteria were determined according to research objectives and research questions. Based on this standard, the text content was classified, and meaningful statements were extracted and coded. Interviewers read and analyzed the semantic units carefully, and distinguished and summarized the theme. Researchers also looked for the relationship between subjects, and formed a theme group. This cycle continued until saturation (no new themes or subthemes were present) [33].

### Quality Control

Before the interviews, the researchers received training in qualitative research, read a large number of relevant literature and books, and learned the analytical methods of qualitative research. The interviewers were involved in the obstetrics clinic as student nurses. As a research tool, researchers always remained neutral. Interviewers truthfully recorded the information provided by interviewees and analyzed their body language and facial expressions. Two researchers with training independently analyzed and discussed the data until the coding information reached a consensus. In the process of data analysis, researchers paid attention to the use of suspension to avoid interference caused by researchers. This study has been reported according to the requirements of the Consolidated Criteria for Reporting Qualitative Research (COREQ) checklist for qualitative studies [34].

### Ethical Considerations

Before each interview, the interviewee was given an explained on the research objectives, methods, expected benefits, and potential risks. Interviewers informed interviewees that relevant information would be strictly confidential. Interviewees could choose to accept or refuse participation in the study, and they could withdraw from the study at any time during the interview. During the interview, interviewees were told that they could refuse to answer any questions that they did not want to answer. Interviewees voluntarily participated in the interviews. In addition, interviewees were told that the content would be used only for scientific research. The interview information was coded, and the researchers did not compromise the privacy of the interviewees.

### Ethics Approval

This study was approved by the Ethics Committee of Capital Medical University (batch number: Z2019SY037) and the Ethics Committee of the hospital conducting the interviews (batch number: 2019-P2-204-02).

## Results

### Design

#### Theme 1: User-Friendly Interface Design

Pregnant women expected clear logic between modules on the app interface and human-computer interaction.

Apps should have logic. What are the second-level interface and third-level interface after opening app? This hierarchy and interface framework should be clear. Make sure the entry and return routes are clear, and then the modules are clear.

Participant #1

Categorize weight management, diet and exercise so that they can be easily seen on the home page. As clear as the app module of hospital appointment. Don't make me look for it, because it's too hard to look for it. A lot of apps these days are really annoying.

Participant #4

Hopefully the app doesn't lag, is smooth to use and doesn't have too many ads.

Participant #6

App can be divided into modules, like what's this, what's that. And when it updates content, don't always change the location.

Participant #7

The app guidelines should be clear. For example, interface modules can be divided into early pregnancy, middle pregnancy and delivery, which are mainly practical and simple.

Participant #10

#### Theme 2: Rich Functionality

Pregnant women hoped that the health management apps they use would cover the common functions of blood glucose management during pregnancy, so as to minimize the use of multiple apps.

Now everyone has several apps in their mobile phones. I hope that in the future there will be an app that contains all pregnancy programs, as convenient as possible.

Participant #4

If the software can provide a one-stop service and integrate these commonly used functions together, I will use only one app. I think it will be much better.

Participant #11

### Functional Requirements

#### Theme 1: Personalized Customization

Pregnant women hoped to obtain personalized recipes customized by medical staff according to their own dietary preferences in mHealth apps, and hoped that these apps have a complete food bank and that they can search for the glycemic index of foods in the apps, so that they could flexibly carry out dietary replacement to reduce their dietary decision confusion.

I've always fantasized about an app that recommends foods I love. I don't have to think about matching my diet myself. It's all done for me, so I just follow the app. But only if I can choose what I like to eat, or if the app removes several ingredients I don't like to eat from the recipe. It would be nice if the app had a search function. For example, if I want to eat strawberries, I can see what the glycemic index of strawberries is and whether it's recommended for me to eat them.

Participant #1

I find it convenient to have recommended recipes in the app so I don't have to think about whether the food is edible or not. It's not realistic for me to follow the recipes exactly, but I'm free to mix and match, as long as the total calories are right, and I think it's better.

Participant #4

In addition, some pregnant women also expressed a need for exercise customization.

I hope the app can let me choose the exercise I want to do every day. For example, today I want to do yoga, and the app can calculate how long I need to do yoga according to my current weight and calorie intake to reach the goal of burning all calories.

Participant #4

#### Theme 2: Accurate Information Support

As the pace of work and life is accelerating, some pregnant women stated that popular science articles recommended by mHealth apps should have attractive titles, be relevant to them, and be more accurate.

At my age, when I have to juggle work and family, I really don't have much free time. For popular science articles, the content is already boring, and if the volume is any longer, users will have no interest or time to read it.

Participant #1

I hope that the app can set up a function of a prenatal assistant to tell me what I should do at different gestational weeks and whether I should have an empty stomach, etc. This is my favorite feature.

Participant #1

The app should recommend relevant contents for pregnant women based on their individual weight and test values. I think this will be better, because I think people are extremely busy nowadays, especially when they have children.

Participant #4

We hope that the app can push suitable exercise types for pregnant women according to their gestational weeks.

Participant #7

If it's a video, it can be put in the appropriate module and I'll pay attention to it when I get to that stage of pregnancy. For example, if it's about breastfeeding, it'll be put in the third trimester and I'll click on it when I'm in labor.

Participant #10

Only when the title of an article is clear and relevant to me will I read it. For example, if I'm eight weeks pregnant, what do I need to be aware of at this stage? I might click on it. But if it's about what to eat and control, I'm probably not going to read it.

Participant #11

#### Theme 3: Interactive Design

Pregnant women hoped that mHealth apps have the function of setting daily calorie and exercise goals. After the pregnant women input their own diet, exercise, and blood sugar monitoring data, the system should provide immediate feedback and professional feedback according to their health management standards, so that the pregnant women can make self-adjustments to achieve their daily management goals.

I hope the app can record the number of steps on the day, and tell me whether the amount of exercise today meets the standard, so as to give me an evaluation.

Participant #1

If I have a problem with my blood sugar, I may check my blood sugar every once in a while and record the result of each time on the app. Then the application platform will give feedback based on my data and tell me what I need to pay attention to in the next step. I think it would be nice to have that kind of feedback.

Participant #4

The app can score me according to my exercise level every week, and then give me an adjustment plan, which forms a closed loop from input, scoring and feedback.

Participant #10

#### Theme 4: Practical Tool Support

Pregnant women hoped that mHealth apps can be used as tools to assist them in self-management and that these apps can record their steps to help them know whether their exercise meets the standard, or can remind and motivate them to exercise.

Although I am in a first-tier city like Beijing, there are few pregnant women around me who do yoga or swim. Especially when you're expecting your first child, families are very cautious. The elders in the family are so ingrained in their beliefs that they may not be comfortable with their children doing yoga during pregnancy, and most would probably prefer to stick to walking during pregnancy. Therefore, it is sufficient for the app to record the number of steps of motion.

Participant #5

For example, I can punch in the app to record my steps today.

Participant #7

In addition, pregnant women hoped that mHealth apps have the function of weight recording to show the trend of their own weight change and that these apps can automatically compare the actual weight increase of pregnant women with the recommended weight increase range to determine whether the weight gain is reasonable, so as to help pregnant women manage their weight during pregnancy.

The app needs to have the function of weight graph, through which we can see the recent trend of pregnant women's weight. I remember when I was pregnant with my first child, there was an app that told me how much weight I needed to gain in my current gestational week.

Participant #4

I am using an app for weight monitoring. I need to input my weight into the app every day to observe the change of my weight.

Participant #9

In addition, some pregnant women hoped that mHealth apps can be used as practical tools to assist doctors in pregnancy management.

The app itself is also a tool to help doctors manage pregnancy health for pregnant women. Since everyone is different, doctors will definitely need to manage pregnancy according to their different physical conditions. It would be better if the app could be linked to hospital records and checklists.

Participant #6

#### Theme 5: Visual Presentation

Some pregnant women stated that they do not have time to watch live broadcasts because of the fast pace of life. They hoped that the information on mHealth apps can be presented in the form of cartoon pictures, risk assessment forms, recorded videos, and short videos, so as to obtain relevant information more quickly.

The recommended article can be in the form of text with cartoon pictures, or it can be one of those risk self-assessment scales for pregnant women to rate their recent status. If the risk score is higher than a certain point, the woman has gestational diabetes.

Participant #10

The live broadcast lasts a long time, and I may not be able to listen to it, because the pace of life is fast now, and life is so busy every day. Let alone live broadcast, I may skip to watch the recorded broadcast.

Participant #5

I think recording is better than live broadcasting. Medical staff can put the video of the lecture in the corresponding module. If I had time, I would listen.

Participant #10

I may not have time to watch the health education live broadcast, if there is a replay, I will watch it. It's better to put subtitles on the video because it's slow and I tend to skip to the subtitles or just read the document.

Participant #6

If experts' lectures are made into some short videos, I may watch them at any time.

Participant #4

#### Theme 6: Convenient Professional Support

Some pregnant women hoped that mHealth apps can provide the online consultation function of experts, so that it is more convenient to contact experts and solve some minor problems that are not too urgent, in order to avoid the cost of time and energy from the round trip to the hospital.

It's more difficult for pregnant women to get to the hospital. If I have any minor questions, I would like to consult one of the experts while they are giving an online lecture. I hope the app can provide free online consultation.

Participant #1

Pregnant women may have some simple problems, but it is not convenient to come to the hospital for registration. It would be better if the app could provide online consultations with doctors. Doctors can choose to reply to pregnant women's information when they have time, there is no rush to reply immediately.

Participant #4

It would be better if pregnant women had some problems that did not need to go to the hospital and could be answered online at a lower cost than going to the hospital.

Participant #9

#### Theme 7: Peer Support

Some pregnant women hoped to set up a function like WeChat Moments or a forum, in order to facilitate access to other pregnant women who have encountered problems and facilitate the exchange of experiences between pregnant women, so as to better deal with the problems of pregnancy.

When I was first pregnant, I went to the forum to see what other people were like when they were pregnant. Many people would share their early pregnancy experiences on the forum, such as problems with their checklists.

Participant #1

If I have some problems, I will look at other users' posts to see how they solve the problems. For example, I have a tummy ache at the early stage of pregnancy, so I will search for users who have the same experience on the Internet. If most users say this is normal, it means that tummy ache is ok.

Participant #8

#### Theme 8: Reasonable Reminder Function

Pregnant women hoped to set reminders according to their own needs. Excessive reminders can disturb pregnant women.

We may not always want to record our diet every day. I hope the app can remind me when I forget to fill in.

Participant #7

I hope the app has a reminder function, such as when I should do activities, when I should drink water, timely reminding me will be better.

Participant #10

I think it is still necessary to set reminders, because the pace of work and life is fast now, pregnant women cannot remember everything every day.

Participant #14

However, some pregnant women believed that an app’s reminder function would cause some trouble, so they had little demand for the reminder function.

Because there are too many reminders in the app, I don't set any reminders in all my apps. I turn them off. If I want to keep track of something important, I'll set up a reminder myself.

Participant #9

#### Theme 9: Appropriate Maternal and Infant Auxiliary Function

Some pregnant women wanted to add mother-baby support tools to mHealth apps to make it through the pregnancy better.

I hope the app can tell me the growth of my baby, so that I can know the development of the baby. In addition, I hope the app can tell me what changes a pregnant woman will have during pregnancy and what changes are normal.

Participant #1

I think the app can add a function to record the gestational weeks.

Participant #2

I have heard that some apps can record contractions and fetal movements. Is it possible to add these functions?

Participant #3

I think there should be a keyword search function, I can query relevant articles, just like Baidu, I want to know can be found in it.

Participant #9

#### Theme 10: Differentiated Incentive Function

Different pregnant women had different views on the redemption function of points. Some thought that it would be better if the points can be exchanged for items they need, while some thought that it has no incentive effect on them.

It is possible to get points by checking in, posting, reading articles, ranking points, and exchanging points for small things, etc. Some people may prefer this function.

Participant #5

I think it would be nice if I could exchange my accumulated points for something to use during pregnancy or in the future.

Participant #6

I don't like those things very much. They are not very useful. I sometimes check to see how many points I have, but I don't think the points are of any use to me.

Participant #7

## Discussion

### Principal Findings

From the perspective of users, this study discussed the views and function requirements of pregnant women at high risk of GDM with regard to the development of mHealth apps for preventing GDM, which can provide a reference for app developers to develop apps in line with users’ usage habits in the future. In this study, pregnant women hoped that when using apps for management, they could receive personalized health management strategies according to their different stages of pregnancy and could receive immediate feedback on their daily management, helping them make adjustments. When pregnant women encountered difficulties in self-management, they hoped to obtain expert and peer resources in the apps to provide them with social support. In addition, pregnant women hoped that developers and professionals would consider the need for quick and accurate information acquisition when designing mHealth apps and would develop user-friendly apps. They stated that the content should be visual and attractive, and should provide both valuable and accurate information to meet the needs of information support.

### Comparison With Prior Work

This study found that it is of great significance to formulate corresponding personalized management strategies for pregnant women based on the results of their health assessment, which is similar to the results of previous studies [35-37]. A research report pointed out that women were dissatisfied with the limited response of apps, and hoped that the apps could specifically analyze the characteristics of users and provide different solutions [38]. The biggest difference between personalized apps and other apps is that personalized apps can provide personalized services for pregnant women in different stages of pregnancy [39]. Studies have shown that personalized features in apps could improve users’ compliance with lifestyle interventions, which is one of the most common intervention strategies in mHealth apps [40].

Interactive function is considered to be the most popular function in mobile medical apps [41]. The biggest advantage of interactive apps is that they can formulate targeted behavior change plans for users, continuously monitor the behavior characteristics of users, and then give targeted feedback according to the implementation of users’ behaviors, which can motivate users to complete behavior changes [42]. Interactive design strengthens the connection between the user and the mobile app, providing psychological support for pregnant women [43]. In this study, pregnant women hope that the apps could compare the health information of pregnant women with the recommended behavioral goals, and provide feedback instantly for pregnant women to help them make timely behavioral adjustments, which is consistent with previous research results [44-46]. Some studies have used telephone or WeChat groups to intervene in pregnant women at high risk of GDM [47,48]. However, it is difficult to achieve instant feedback between doctors and patients, and the information of telephone and WeChat groups is also easily forgotten and covered. Therefore, it is important to design an app to prevent gestational diabetes. There is also a study using an app that counts low glycemic index function to provide dietary guidance to obese pregnant women, but this app has a single function and is designed for dietary guidance only, which cannot achieve the goal of interaction, resulting in an insignificant intervention effect [49].

Due to time constraints, most working pregnant women hoped apps would accurately push information related to themselves. Previous studies have found that providing users with personally relevant information was an important factor in promoting the use of mHealth programs [50]. In addition, Naughton et al [51] found that pushing personalized and relevant information could increase the value of contents and prevent users from falling out of mHealth management. In a qualitative study of Saudi women, it was found that due to social and cultural restrictions, some obese women could not do enough physical exercise outdoors [52]. Interviewees hoped that apps could recommend exercise methods that meet actual conditions for users and provide advice according to users’ preferences.

Most pregnant women hoped that information could be in visual forms, such as illustrations and videos, to increase attractiveness. Presenting content in a visual way can help women obtain more information in a short period of time to meet women's needs for pregnancy knowledge. Previous studies have pointed out that multimedia could adapt to the learning styles of different individuals, enable pregnant women to take the initiative in learning, and encourage pregnant women to actively study [53,54]. Some studies have pointed out that the memory acquired by watching videos was stronger than that acquired by browsing text or pictures [55]. This study found that in terms of the video presentation form, pregnant women were more inclined to short videos or recorded videos. Therefore, when designing apps, developers should use a visual method as much as possible to present a variety of health information and educational resources for pregnant women to meet their needs for information support.

During the daily management of pregnancy, pregnant women will obtain decision-making information from different channels. Studies have shown [56] that pregnant women tend to trust professional decision support. Professional support provided by medical staff can meet the needs of pregnant women in pregnancy health care, which relieves anxiety and uncertainty of women during pregnancy [57,58] and helps pregnant women establish a healthy lifestyle [59]. When professionals cannot provide timely and effective feedback, pregnant women usually seek help from relatives and friends or through the internet. However, due to the poor quality of advice from various parties, pregnant women often get confused and make wrong decisions, which is not conducive to health management [60]. This study found that pregnant women hoped to obtain professional feedback from apps when they encountered problems or in the process of daily health management. This is similar to previous studies by Edwards et al [61] and Lau et al [43]. Previous studies have shown that when patients were unable to communicate effectively with experts, they felt bored and useless to use an app, resulting in a low utilization rate [36]. Therefore, when developing apps in the future, professional support functions should be added. In addition, when apps are actually used to manage the health of pregnant women, managers should coordinate the workload distribution of medical staff and include answering questions from pregnant women into their work scope, providing users with timely and effective professional support.

In addition to professional support, peer support also plays an important role in the formation and maintenance of patients’ self-management abilities. Peers can communicate with each other on their own experience, attitudes, and concepts, giving relevant suggestions to each other. Pregnant women in this study hoped that they could share confusion, emotion, and experience with peers who had similar needs and common goals through an app; thus, these women can obtain information and emotional support. This is similar to the findings of McDonald et al [62]. Studies have shown that peer support could overcome loneliness, powerlessness, and stress in pregnant women and increase maternal self-efficacy. Emotional support and exchange of experiences with peers are more acceptable to pregnant women. In addition, pregnant women can gain motivation from peer support to use an app [63]. Previous studies have found that providing a communication platform for users in a GDM prevention app could allow users to exchange their experiences and deal with problems during pregnancy, motivate users to achieve their expected goals, and increase the frequency of usage [64]. Therefore, apps should provide a module for pregnant women to communicate with their peers, allowing them to learn problem-solving skills through mutual assistance and to enhance psychological support.

Although some pregnant women in this interview study believed that the point reward function was of little significance, research has proven that the introduction of game mechanics could increase the user’s sense of achievement and could cause the user to put more effort into accomplishing goals. Cafazzo et al [65] incorporated a gamification mechanism when designing a diabetes blood sugar management program. When young patients regularly checked their blood glucose, they could obtain corresponding points. These points could be redeemed for iTunes codes to purchase music and applications. The frequency of blood glucose monitoring increased by nearly 50% in patients using the apps. Ekezie et al [64] provided a virtual map for gestational diabetes mellitus patients in his app to improve the interest of patients’ exercise, and set a step ranking list to encourage users to achieve exercise goals through competition, so as to increase user efficiency. It is suggested that when developing gestational diabetes prevention apps in the future, the gamification and reward mechanisms should be improved according to users’ needs. Furthermore, more attractive methods should be set up to better assist patients in performing self-management, as well as raise their enthusiasm for using these apps.

### Strengths and Limitations

Previous research has mostly focused on the user’s experience and evaluation of developed apps after using them, and few users have participated in the design of apps, leading to poor user stickiness. In this study, we conducted deep interviews on the preferences and functional needs of mHealth apps among people at risk for gestational diabetes, and the findings are important to guide the development of future interventions and other pregnancy health apps. In addition, the strength of this study is that the data analysis was performed by two researchers and information was continuously validated during the data analysis process. One of the researchers was the interviewer and the other was not involved in the interview. Therefore, in the process of data analysis, the two researchers had a different understanding of the existing data. Then they discussed and finally reached a more objective and unified opinion, and provided the analysis results to the participants to make their opinions expressed fully and correctly.

This study has several limitations. First, this study only interviewed 15 pregnant women in the same hospital in Beijing, China. Regardless of the fact that the sample size of this study reached saturation, the method of maximum difference sampling was adopted to select the research objects as much as possible. However, no interviewees from rural groups were included; thus, the interview results may not reflect the diverse needs of all pregnant women, making it difficult to generalize the findings to women in rural areas or different cultural situations. Before the formal interview, interviewers trained and practiced to improve their interviewing skills. However, as interviewers were conducting interviews for the first time, their interviewing experience and skills were limited, which may have affected the comprehensiveness of data collection. In addition, interviewers conducted interviews as medical interns, which made some interviewees reluctant to express their true feelings and needs, leading to a potential impact on data collection.

### Conclusion

mHealth apps can provide new tools for the health management of people at risk for gestational diabetes. Before the development of a gestational diabetes prevention app, this study deeply collected the views and functional requirements of mHealth app development from gestational diabetes risk groups. The results can provide valuable information for the future development of mHealth apps related to the prevention of gestational diabetes that meet users’ needs, and can provide a reference for the design of intervention content in the future. In addition, the findings can provide a reference for the development of other types of apps during pregnancy.

## Abbreviations

GDM: gestational diabetes mellitus
mHealth: mobile health
UCD: user-centered design
